# Poly(ethylene Glycol) (PEG)–OligoRNA Hybridization to mRNA Enables Fine‐Tuned Polyplex PEGylation for Spleen‐Targeted mRNA Delivery

**DOI:** 10.1002/smsc.202300258

**Published:** 2024-02-22

**Authors:** Miki Suzuki, Yuki Mochida, Mao Hori, Akimasa Hayashi, Kazuko Toh, Theofilus A. Tockary, Xueying Liu, Victor Marx, Hidetomo Yokoo, Kanjiro Miyata, Makoto Oba, Satoshi Uchida

**Affiliations:** ^1^ Medical Chemistry Graduate School of Medical Science Kyoto Prefectural University of Medicine 1‐5 Shimogamohangi‐cho, Sakyo‐ku Kyoto 606‐0823 Japan; ^2^ Department of Advanced Nanomedical Engineering Medical Research Institute Tokyo Medical and Dental University (TMDU) 1‐5‐45 Yushima, Bunkyo‐ku Tokyo 113‐8510 Japan; ^3^ Innovation Center of NanoMedicine (iCONM) Kawasaki Institute of Industrial Promotion 3‐25‐14 Tonomachi, Kawasaki‐ku Kawasaki 210‐0821 Japan; ^4^ Department of Materials Engineering Graduate School of Engineering The University of Tokyo 7‐3‐1 Hongo, Bunkyo‐ku Tokyo 113‐8656 Japan; ^5^ Department of Pathology Kyorin University School of Medicine 6‐20‐2 Shinkawa, Mitaka‐shi Tokyo 181‐8611 Japan

**Keywords:** intravital imagings, mRNA deliveries, mRNA engineerings, polyplexes, tissue targetings

## Abstract

Organ‐selective targeting of mRNA polyplexes has been rarely explored despite the substantial potential of polymer‐based systems in mRNA delivery. In this study, spleen‐selective delivery of polyplexes is achieved by employing mRNA engineering to coat them with poly(ethylene glycol) (PEG). In this approach, mRNA is hybridized with PEGylated complementary RNA oligonucleotides (PEG–OligoRNAs), followed by the addition of linear poly(ethyleneimine). In this method, it is ensured that nearly all added PEG strands bind to the polyplexes, thereby enabling precise control of PEG amounts on the surface. Following systemic injection into mice, non‐PEGylated polyplexes yield robust protein expression in the lung and spleen. Intriguingly, adding a small number of PEG–OligoRNAs drastically reduces protein expression efficiency in the lung while preserving it in the spleen, realizing spleen targeting of mRNA polyplexes. Furthermore, PEGylated polyplexes demonstrate their potential utility in mRNA vaccination. In mechanistic analyses, non‐PEGylated polyplexes immediately agglomerate in the blood and deposit in the lung. Coating polyplexes with a small amount of short PEG effectively prevents these processes. Notably, even slight changes in PEG amounts and lengths dramatically impact the physicochemical properties and biological functionalities of the polyplexes, emphasizing the benefits of an mRNA engineering‐based approach for fine‐tuning polyplex PEG coating.

## Graphical abstract

Controlled poly(ethylene glycol) (PEG)‐coating of mRNA polyplexes is accomplished through mRNA engineering. This involves hybridizing mRNA with PEGylated complementary RNA oligonucleotides and mixing it with polycations to prepare PEGylated polyplexes. Upon systemic injection in mice, polyplexes with the optimal number and length of PEG strands achieve spleen‐selective mRNA delivery by preventing agglomeration in the blood and lung deposition.

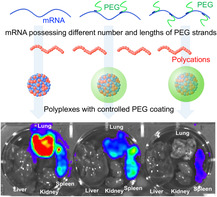

## 1 Introduction

The success of mRNA vaccines for coronavirus disease 2019 (COVID‐19) is driving extensive research and development of mRNA therapeutics across various medical fields, encompassing cancer immunotherapy, protein replacement therapy, and genome editing.^[^ ^1^, ^2^, ^3^, ^4^ ^]^ These applications demand organ‐selective mRNA delivery systems to achieve maximum therapeutic benefits while minimizing safety concerns related to off‐target mRNA expression. Systemic administration presents a means to target various organs and cancers via the bloodstream circulating throughout a body. For selective tissue targeting, the conventional approach involves introducing targeting ligands. However, this often necessitates intricate nanoparticle designs^[^ ^5^, ^6^, ^7^ ^]^ and can escalate economic costs when employing antibodies or other elaborate ligands. In contrast, recent studies have successfully altered the organ selectivity of systemically delivered mRNA nanoparticles by simply modulating the physicochemical properties of the nanoparticles.^[^ ^8^, ^9^, ^10^ ^]^ For instance, adding cationic lipids to ionizable lipid‐based nanoparticles (LNPs) redirects LNPs from the liver to the lung, whereas incorporating anionic lipids confers spleen selectivity to the LNPs.

While organ‐selective mRNA targeting has been well documented in LNPs, it has not been extensively explored in polymer‐based mRNA nanoparticles, specifically polyplexes. Polyplexes offer diverse functionalities for stable extracellular mRNA packaging, efficient endosome escape, and controlled intracellular release, making them a promising class of mRNA delivery systems.^[^ ^11^, ^12^, ^13^, ^14^, ^15^ ^]^ In contrast to LNPs, which inherently target the liver, cationic polyplexes prepared from polycations and nucleic acids tend to accumulate in the lung.^[^ ^16^, ^17^ ^]^ Mechanistic analyses elucidate that the lung accumulation of polyplexes results from their rapid agglomeration in the blood, leading to clotting in the lung capillaries.^[^ ^18^, ^19^, ^20^ ^]^ Thus, preventing polyplex agglomeration may redirect their selectivity toward organs other than the lung, reducing safety concerns associated with lung embolism.

PEGylation of polyplexes effectively prevents their agglomeration in the blood,^[^ ^18^, ^19^, ^20^ ^]^ but achieving precise control of PEG density on the polyplex surface is challenging despite its potential impact on in vivo behavior and functionalities. The conventional method of blending non‐PEGylated and PEGylated polycations^[^ ^21^, ^22^, ^23^ ^]^ encounters difficulties in controlling the actual binding number of non‐PEGylated and PEGylated polycations to mRNA, as both polycation components can exist in free and mRNA‐binding forms. The feeding ratio of non‐PEGylated and PEGylated polycations to mRNA may not accurately correspond to their actual binding ratio when they exhibit different affinities for mRNA.

**Figure 1:**
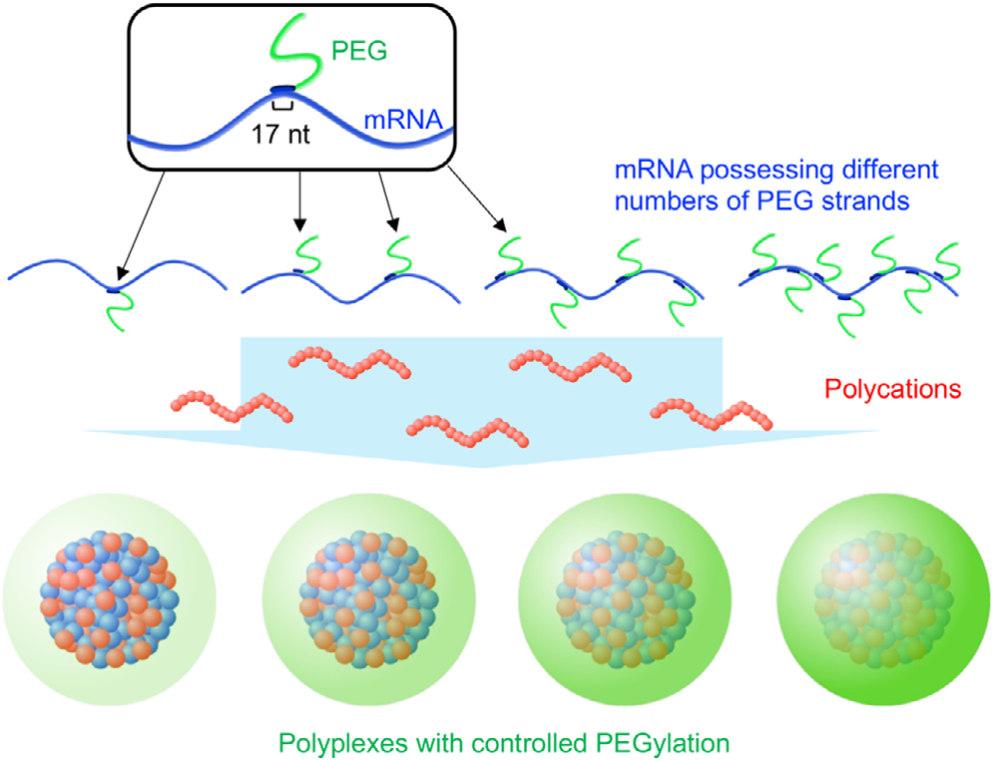
Polyplex PEGylation using polycations and mRNA hybridized with a defined number of PEG–OligoRNAs.

Alternatively, we adopted an mRNA engineering approach, leveraging the RNA hybridization mechanism as a more reliable strategy for polyplex PEGylation. In this approach, mRNA was hybridized with PEGylated complementary RNA oligonucleotides (PEG–OligoRNAs) and then mixed with polycations (**Figure** **1**). This method ensures that nearly all the added PEG strands bind to the polyplex, allowing easy and precise adjustment of PEG density on the polyplex surface by varying the amount of PEG–OligoRNAs. Utilizing this system, we created a library of polyplexes with varying PEG quantities and lengths. Intriguingly, hybridizing a small number of PEG–OligoRNAs with short PEG lengths enabled spleen‐selective delivery of mRNA polyplexes following systemic injection into mice by preventing their agglomeration and subsequent lung deposition. Conversely, increasing the number of PEG–OligoRNAs or extending the PEG lengths from the optimal configuration substantially impaired mRNA delivery efficiency in the spleen. Even minor alterations in the PEG‐coating status significantly influenced the physicochemical properties and biological functionalities of the polyplexes, underscoring the significance of our original approach for finely tuning PEG coating on the polyplexes.

## 2 Results and Discussion

### 2.1 mRNA PEGylation and Polyplex Preparation

**Figure 2:**
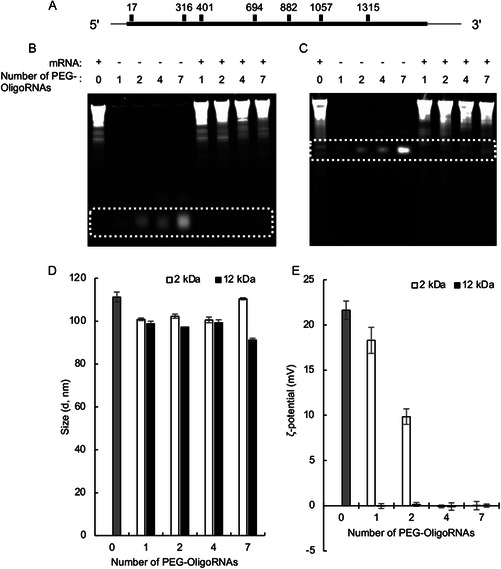
Preparation of PEGylated mRNA and polyplexes. A) Positions of PEG–OligoRNAs along an *fLuc* mRNA strand. The numbers indicate the distance between the first base of PEG‐OligoRNAs and the start codon (nt). B,C) Gel electrophoresis of mRNA, PEG–OligoRNAs, and mRNA hybridized with PEG–OligoRNAs. B) PEG_2k_–OligoRNAs, C) PEG_12k_–OligoRNAs, and D) sizes and E) ζ potentials of the polyplexes, *n* = 3. Data are presented as the mean ± standard deviation (SD).

We designed seven PEG–OligoRNAs to target various positions in the coding sequence of *firefly luciferase* (*fLuc*) mRNA, which consists of 1929 nucleotides (nt) (**Figure** **2**A). We avoided targeting RNA regions predicted to have secondary structures by software. The hybridization length of the PEG–OligoRNAs was fixed at 17 nt, following our prior study that investigated the impact of complementary RNA lengths on mRNA translational efficiency after hybridization.^[^ ^24^ ^]^ After hybridization with 23 nt or longer RNA, mRNA translational activity decreased. However, it remained constant after 17‐nt RNA hybridization. A subsequent mechanistic study suggested that endogenous helicase activity detaches complementary RNA from mRNA inside cells during 5' cap‐dependent translational processes, facilitating further translation.^[^ ^25^ ^]^ We conjugated a 2 or 12 kDa PEG strand to the 5' end of the 17 nt RNA oligonucleotide, naming the resulting PEG–OligoRNAs as PEG_2k_–OligoRNA and PEG_12k_–OligoRNA, respectively. mRNA and each sequence of PEG–OligoRNA were mixed at a 1:1 molar ratio, followed by heating and gradual cooling to promote hybridization. Subsequent gel electrophoresis revealed that unhybridized PEG–OligoRNAs were almost undetectable, confirming the successful hybridization of nearly all PEG–OligoRNAs to mRNA (Figure 2B,C). This result is consistent with our previous studies, which demonstrated an almost 100% hybridization ratio of mRNA and PEG–OligoRNAs.^[^ ^26^, ^27^ ^]^

The PEGylated mRNA prepared in this manner was then mixed with linear poly(ethyleneimine) (LPEI) to formulate polyplexes. The ratio of amino groups in LPEI (N) to phosphate groups in PEGylated mRNA (P) (N/P ratio) was set to 4. This N/P ratio ensures the successful binding of all mRNA to LPEI, as confirmed by the gel electrophoresis (Figure S1, Supporting Information). In dynamic light scattering (DLS) measurements, the polyplexes had a size of approximately 100 nm with a polydispersity index below 0.15, irrespective of the presence and number of PEG–OligoRNAs and the length of PEG strands (Figure 2D, Table S1, Supporting Information). The ζ potential of the polyplexes decreased as the number of PEG_2k_‐OligoRNAs hybridized to mRNA increased, while the hybridization of a single PEG_12k_–OligoRNA was sufficient to neutralize the positive charge of LPEI/mRNA polyplexes (Figure 2E). Notably, even a minor change in the number of PEG_2k_–OligoRNAs drastically impacted on the ζ potential. In detail, the ζ potential of non‐PEGylated polyplexes (+21.6 mV) showed minimal change after hybridization with 1 PEG_2k_–OligoRNA but largely decreased to +9.9 mV after hybridization with 2 PEG_2k_–OligoRNAs and eventually became nearly neutral after hybridization with 4 PEG_2k_–OligoRNAs.

### 2.2 Polyplex Functionalities in Cultured Cells

**Figure 3:**
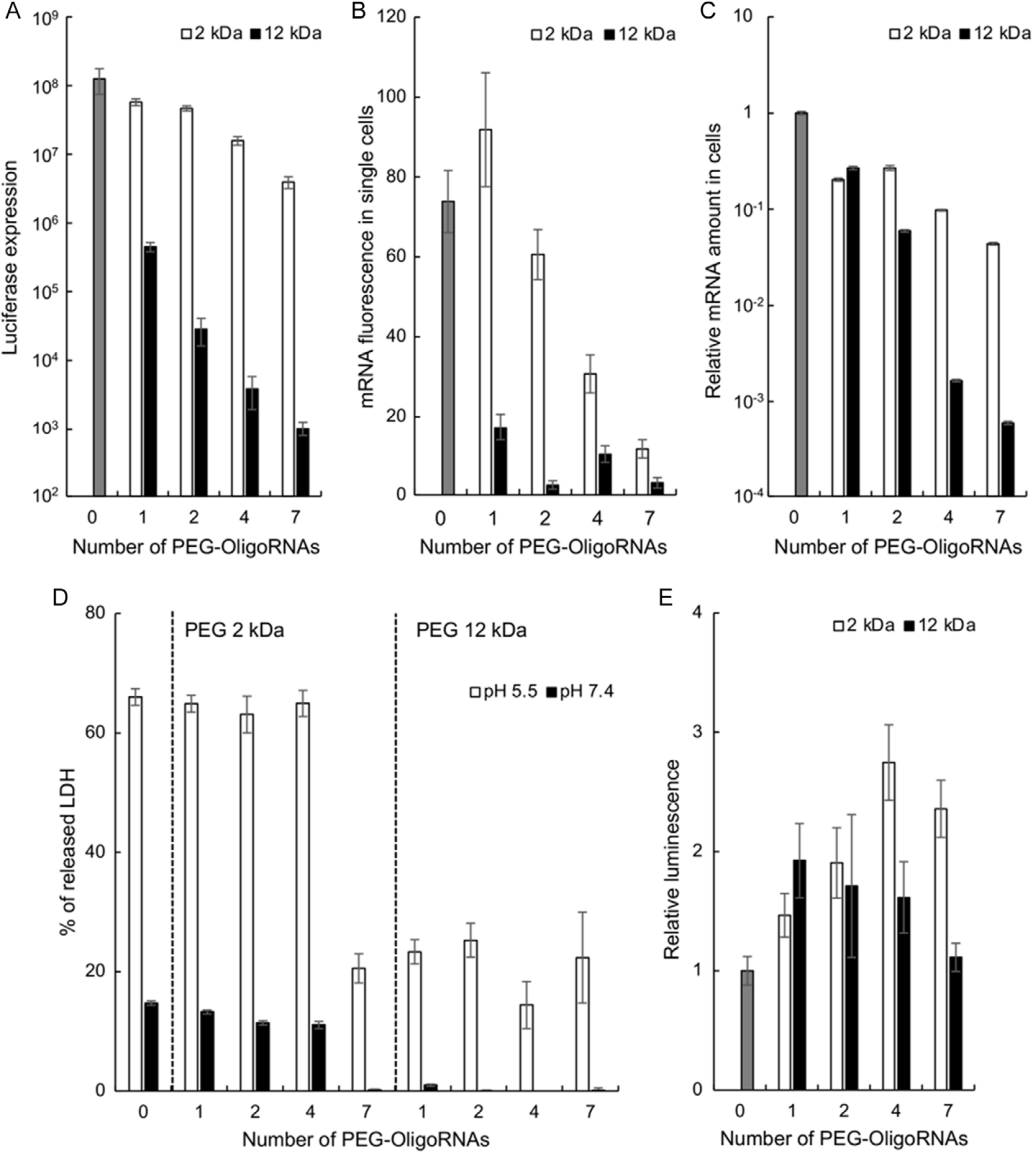
In vitro biological functions of the PEGylated polyplexes. A) fLuc expression efficiency after 24 h of polyplex treatment, *n* = 6. B) Cellular uptake efficiency of Cy3‐labeled mRNA, evaluated by flow cytometry after 1 h of polyplex treatment, *n* = 3. C) Cellular uptake efficiency of mRNA evaluated by qPCR after 24 h of polyplex treatment, *n* = 3. D) Destabilization of the cell membrane at an endosomal pH of 5.5 and a physiological pH of 7.4, *n* = 6. Lactate dehydrogenase (LDH) amount relative to a lysis–buffer‐treated control is shown. E) Cell‐free fLuc translation efficiency, *n* = 7. Data are presented as the mean ± standard error of the mean (SEM).

The mRNA delivery capability of PEGylated polyplexes in cultured cells was evaluated using DC2.4 cells, a murine dendritic‐cell‐derived cell line, after a 24 h treatment with *fLuc* mRNA. PEG introduction reduced fLuc protein expression efficiency with an increase in the length of PEG and the number of PEG–OligoRNAs hybridized to mRNA (**Figure** **3**A). The result in Figure 3A clearly illustrates that even a modest alteration in PEG coating drastically influences the biological performance of the polyplexes. Meanwhile, attachment of 1 or 2 PEG_2k_–OligoRNAs preserved the expression level to more than one‐third of that observed after the treatment of the polyplex without PEG coating. Similarly, polyplex PEGylation demonstrated inhibitory effects on luciferase expression efficiency in lung‐cancer‐derived A549 cells and hepatic‐cancer‐derived HuH‐7 cells, depending on the number and length of PEG strands (Figure S2A,B, Supporting Information). Notably, DC2.4 cells exhibited an approximately tenfold higher level of fLuc expression than A549 and HuH‐7 cells (Figure S2C, Supporting Information).

To gain mechanistic insights, we first evaluated cellular uptake efficiency using mRNA labeled with Cy3, a fluorescent dye. Flow cytometry measurements after 1 h of polyplex treatment revealed that cellular uptake efficiency decreased as the number of PEG–OligoRNAs and the length of PEG increased (Figure 3B, S3, Supporting Information), consistent with the observations for fLuc expression efficiency (Figure 3A and S2, Supporting Information). This fluorescence‐based measurement can detect enzymatically degraded mRNA as well as intact mRNA. The effect of enzymatic mRNA degradation may become critical overtime, leading us to choose an early time point of 1 h for the flow cytometry experiment. To evaluate the total mRNA uptake at a later time point (24 h post‐polyplex addition), we also measured mRNA levels using quantitative polymerase chain reaction (qPCR), which detects mRNA with an intact sequence between primers. In accordance with the results from flow cytometry, qPCR revealed the inhibitory effect of PEG on cellular uptake in DC2.4 cells, which depended on the number and length of PEG chains (Figure 3C).

Next, the endosomal membrane destabilization capability of the polyplexes was evaluated using the cell membrane as a surrogate. In this experiment, DC2.4 cells were incubated with polyplexes at an endosomal pH of 5.5, which destabilized the cell membrane to release LDH. The amount of released LDH reflects the degree of membrane destabilization at the endosomal pH. Adding 7 PEG_2k_–OligoRNAs and 1–7 PEG_12k_–OligoRNAs decreased the LDH release at pH 5.5 compared to that in non‐PEGylated polyplexes, while 1–4 PEG_2k_–OligoRNAs showed negligible effects on LDH release (Figure 3D). This result indicates that PEG chains decrease the efficiency of polyplex endosomal escape as their number and lengths increase. Notably, all tested polyplexes induced LDH release less efficiently at pH 7.4 than at pH 5.5. Such a pH‐responsive membrane destabilization profile may be critical to achieving efficient endosomal escape with minimal cytotoxicity.

Lastly, the protein translational activity of the polyplexes in the intracellular environment was evaluated by measuring fLuc expression from the polyplexes incubated in rabbit reticulocyte lysate, which mimics the intracellular environment. As a result, a series of PEGylated polyplexes with varying numbers and lengths of PEG strands exhibited fLuc translational activity at comparable or higher levels compared to that observed for non‐PEGylated polyplexes (Figure 3E).

It would be meaningful to discuss the binding behavior of PEG–OligoRNAs to mRNA during the delivery to the cells. In this process, the addition of PEG–OligoRNAs significantly reduced cellular uptake efficiency (Figure 3B,C). This result indicates that PEG strands stably bind to the polyplexes in the extracellular environment and inhibit the cellular uptake of the polyplexes. Meanwhile, several results suggest the detachment of PEG–OligoRNAs from mRNA inside the cells. Increasing the number and length of PEG strands concurrently decreased luciferase efficiency (Figure 3A) and intracellular mRNA amount (Figure 3C). These results suggest that the primary reason for reduced luciferase expression in highly PEGylated polyplexes may be the reduced intracellular amount of intact mRNA, while the mRNA is translatable once inside the cells. This is consistent with the experimental finding that highly PEGylated polyplexes showed protein translation efficiency at a level comparable to non‐PEGylated polyplexes in a cell‐free environment mimicking the intracellular environment (Figure 3E). Such efficient translation may require the detachment of PEG–OligoRNAs from mRNA. These results are consistent with our previous reports, wherein mRNA preserves its translational activity after hybridization with complementary RNA modified with cholesterol, PEG, or other functional moieties.^[^ ^24^, ^26^, ^27^, ^28^, ^29^ ^]^ PEG–OligoRNAs might be detached intracellularly via the endogenous helicase activity of the protein translational machinery.^[^ ^25^ ^]^

To obtain further insight into cellular uptake mechanisms, we evaluated the influence of endocytosis inhibitors on luciferase expression efficiency in DC2.4 cells, using chlorpromazine for inhibiting clathrin‐mediated endocytosis, genistein for inhibiting caveolae‐mediated endocytosis, and cytochalasin D for inhibiting phagocytosis and micropinocytosis (Figure S4, Supporting Information). Chlorpromazine reduced luciferase expression by up to tenfold in all tested polyplexes, except for polyplexes with 4 PEG_12k_–OligoRNAs, which showed inefficient luciferase expression even without endocytosis inhibitor treatment. In addition, non‐PEGylated polyplexes and those with 2 PEG_2k_–OligoRNAs received a negative impact from cytochalasin D. Therefore, clathrin‐mediated endocytosis is an essential pathway for cellular uptake in most polyplexes regardless of the number and length of PEG chains, while non‐PEGylated polyplex and polyplexes with a small number of short PEG chains also use phagocytosis or micropinocytosis.

Collectively, reduced efficiency of cellular uptake and endosomal escape may explain the inhibitory effect of PEG on mRNA delivery to cultured cells. Specifically, the masking of cationic charge by PEG (Figure 2E) might inhibit electrostatic interaction between polyplexes and negatively charged cell and endosomal membranes. However, the decrease in ζ potential after PEGylation alone does not fully explain the decrease in fLuc expression efficiency. Indeed, the efficiency of fLuc expression and cellular uptake is substantially different between six formulations of neutrally charged polyplexes, i.e., those loading mRNA with 4 and 7 PEG_2k_–OligoRNAs and 1–7 PEG_12k_–OligoRNAs. Long PEG chains and a dense PEG layer on the polyplex surface may hamper the interaction between polyplexes and the cell membrane.

### 2.3 Systemic Delivery of the Polyplexes

**Figure 4:**
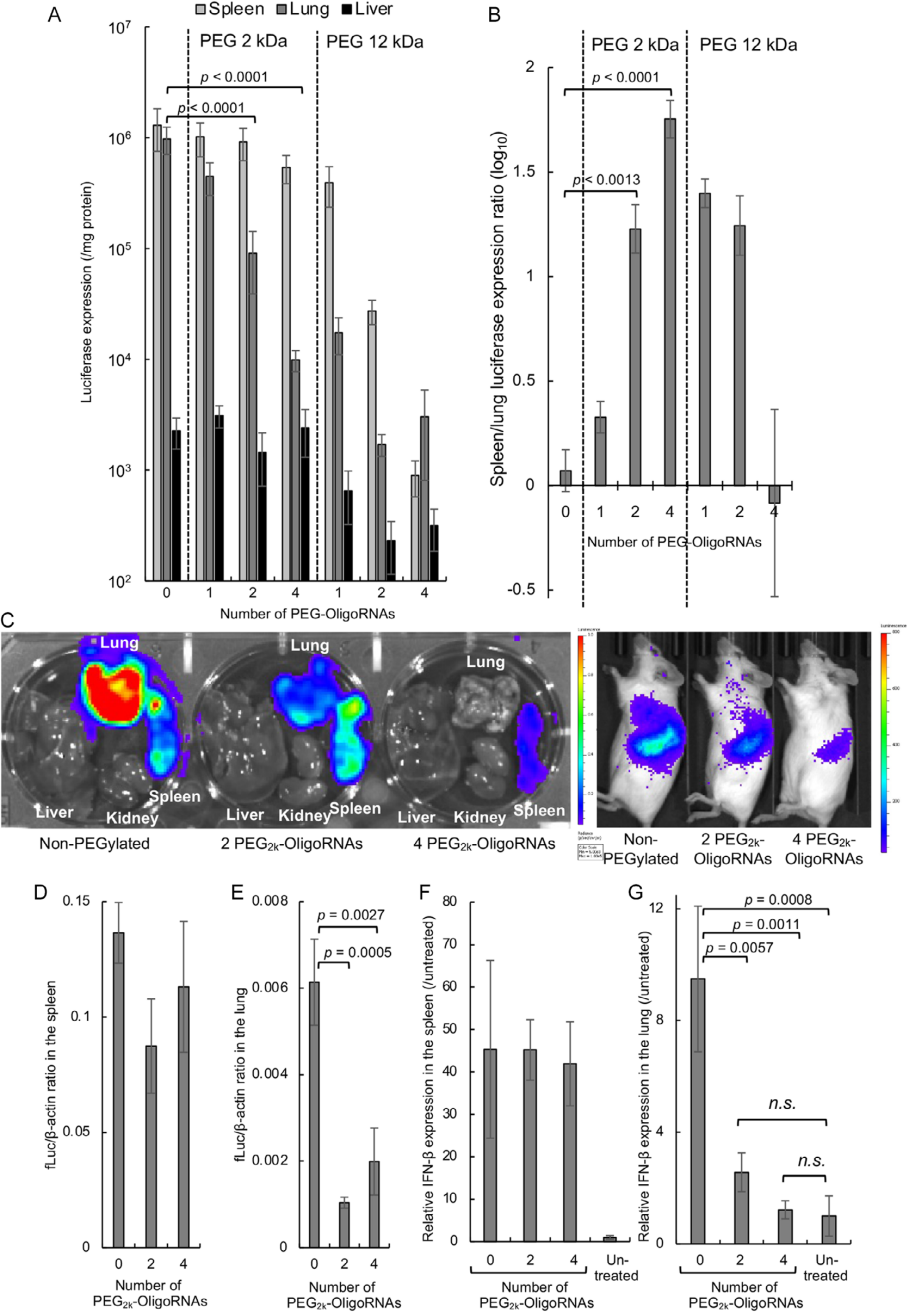
Distribution of protein expression and mRNA after systemic injection in mice. fLuc expression in each organ 4 h after the injection: A) fLuc expression in tissue homogenates; B) fLuc expression levels in the spleen relative to those in the lung: log‐transformed values calculated from the data in A) are shown; C) luminescence imaging of excised organs and living animals. *fLuc* mRNA levels in D) the spleen and E) lung, evaluated by qPCR 4 h postinjection. *IFN‐β* mRNA levels in F) the spleen and G) lung, evaluated by qPCR 4 h postinjection, *n* = 6. Data are presented as the mean ± SEM. Statistical analyses were performed by analysis of variance (ANOVA) followed by Tukey's post hoc test.

The in vivo mRNA delivery efficiency and tissue selectivity of the mRNA polyplexes were assessed following their intravenous injection into mice using fLuc as a reporter. At 4 h postinjection, non‐PEGylated polyplexes exhibited robust fLuc expression in the spleen and lung while showing almost undetectable expression in the liver (**Figure** **4**A). Similar to the in vitro results (Figure 3A), mRNA PEGylation tended to decrease fLuc expression efficiency, especially in the spleen and lung. However, the inhibitory effect substantially varies between these two organs. Compared to non‐PEGylated polyplexes, polyplexes loaded with mRNA hybridized with 2 PEG_2k_–OligoRNAs exhibited nearly equivalent fLuc expression efficiency in the spleen but approximately tenfold lower efficiency in the lung. Similarly, hybridization with 4 PEG_2k_–OligoRNAs reduced fLuc expression efficiency by 2.4‐fold in the spleen but by approximately 100‐fold in the lung. These findings demonstrate that PEGylation of the polyplexes enhances the organ selectivity of fLuc expression in the spleen. This trend is clearly depicted in Figure 4B, which illustrates the ratios of protein expression efficiency in the spleen compared to that in the lung (Figure 4B). The ratio is 1.2 for non‐PEGylated polyplexes, 17 for polyplexes with 2 PEG_2k_–OligoRNAs, and 57 for polyplexes with 4 PEG_2k_–OligoRNAs, highlighting the remarkable improvement in spleen selectivity after PEGylation. Subsequent sections of this study utilize mRNA hybridized with 2 and 4 PEG_2k_–OligoRNAs for functional and mechanistic evaluations, as these formulations exhibited high selectivity and efficiency in fLuc expression in the spleen. This trend is also evident by direct luminescence observation from the extracted organs and living animals (Figure 4C).

To gain mechanistic insights, we assessed mRNA distribution in the spleen and lung by qPCR of *fLuc* mRNA 4 h post‐mRNA injection. The hybridization of 2 or 4 PEG_2k_–OligoRNAs had a minimal impact on mRNA distribution in the spleen compared to that observed after injecting non‐PEGylated polyplexes (Figure 4D). In contrast, mRNA distribution in the lung significantly decreased following the hybridization of 2 or 4 PEG_2k_–OligoRNAs (Figure 4E). The mRNA accumulation profile in the lung may explain the reduced fLuc expression efficiency in the lung after PEG–OligoRNA hybridization (Figure 4A). The mRNA distribution profile is well correlated with the type I interferon (IFN) responses. All formulations increased the levels of IFN‐β transcripts in the spleen, irrespective of the presence and number of PEG_2k_–OligoRNAs (Figure 4F). In contrast, polyplex PEGylation using 2 or 4 PEG_2k_–OligoRNAs significantly reduced IFN‐β induction in the lung compared to non‐PEGylated polyplexes (Figure 4G). The IFN‐β levels observed in the PEGylated polyplex groups were comparable to those in an untreated control.

Upon close examination, the attachment of 2 or 4 PEG_2k_–OligoRNAs did not affect the polyplex accumulation efficiency in the spleen (Figure 4D) but slightly decreased luciferase expression efficiency in the spleen compared to the non‐PEGylated polyplexes (Figure 4A,C). Thus, PEGylation may reduce the efficiency of luciferase expression at the later steps after polyplex accumulation in the spleen, including cellular uptake, endosomal escape, and protein translation. Among these steps, the attachment of 2 or 4 PEG_2k_–OligoRNAs may not affect the efficiency of endosomal escape (Figure 3D) and mRNA translation in the cytoplasm (Figure 3E), according to in vitro experiments. These results suggest that PEGylation might reduce cellular uptake efficiency after accumulation in the spleen.

**Figure 5:**
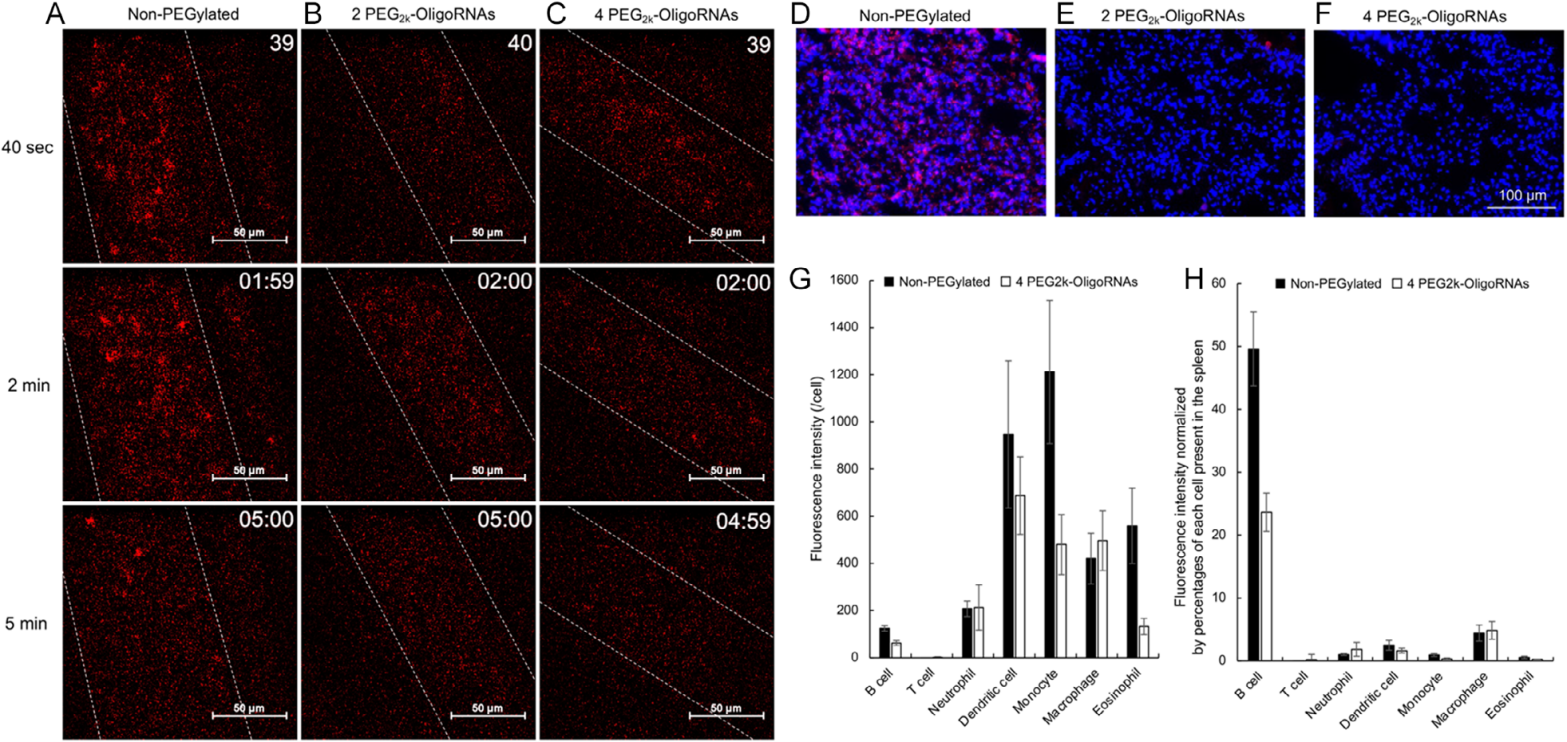
Imaging of polyplexes in mice. Cy5‐labeled mRNA was used to visualize polyplexes. A–C) Intravital real‐time confocal laser‐scanning microscopy (IVRT–CLSM) imaging of blood vessels in the earlobe approximately 40 s, 2 min, and 5 min after intravenous injection. Positions of the veins are shown in the dotted lines. D–F) Fluorescence images of lung tissue sections 30 min after polyplex injection. Red: Cy5‐labeled mRNA; blue: cell nuclei. A,D) Polyplexes without PEGylation. B,E) Polyplexes with 2 PEG_2k_–OligoRNAs. C,F) Polyplexes with 4 PEG_2k_–OligoRNAs. G,H) Types of immune cells taking up the polyplexes in the spleen 4 h post intravenous injection. The uptake efficiency was quantified by flow cytometry. G) The uptake efficiency per cell. H) The uptake efficiency normalized by the percentage of each cell type present in the spleen, *n* = 5. Data are presented as the mean ± SEM.

To explain the difference in polyplex distribution profiles, we directly observed the polyplexes in the blood circulation of live mice using intravital real‐time confocal laser‐scanning microscopy (IVRT–CLSM). mRNA polyplexes were visualized by fluorescently labeling mRNA with Cy5. Non‐PEGylated polyplexes formed several‐micrometer‐sized agglomerates within 1 min after intravenous injection (**Figure** **5**A). This observation aligns with a prior study, which showed the immediate agglomeration of non‐PEGylated plasmid DNA polyplexes in blood circulation.^[^ ^20^ ^]^ In contrast, polyplex PEGylation using 2 or 4 PEG_2k_–OligoRNAs successfully mitigated the agglomeration of the polyplexes in the bloodstream (Figure 5B,C).

Based on previous studies, polyplex agglomeration triggers platelet activation and subsequent lung embolism.^[^ ^18^, ^19^, ^20^, ^30^, ^31^ ^]^ In accordance with these findings, intravenous injection of non‐PEGylated polyplexes labeled with Cy5‐mRNA resulted in the appearance of over a few micrometer‐sized dots in the lung section (Figure 5D), which might represent the clotting of capillaries with the polyplexes. In contrast, polyplex PEGylation using 2 or 4 PEG_2k_–OligoRNAs effectively prevented the deposition of mRNA polyplexes in the lung (Figure 5E,F). Notably, the signal from Cy5 mRNA was almost undetectable in the lung section after the injection of PEGylated polyplexes, even though qPCR detected the presence of mRNA in the lung in these groups (Figure 4E). Fluorescence microscopy might preferentially visualize large agglomerates in non‐PEGylated polyplexes, leaving fluorescence signals from diffused mRNA undetected in the PEGylated polyplex groups. From a safety perspective, both non‐PEGylated polyplexes and PEGylated polyplexes did not induce apparent behavioral abnormalities, lethality, or changes in blood chemistry in mice (Figure S5, Supporting Information). However, previous studies have raised potential safety concerns related to polyplex agglomeration‐induced lung embolism.^[^ ^18^, ^19^, ^20^, ^30^, ^31^ ^]^

While non‐PEGylated and PEGylated polyplexes displayed different behaviors in the blood and lung, they similarly exhibited substantial accumulation in the spleen (Figure 4A–D). To obtain more profound insight into this result, we identified the types of immune cells responsible for the uptake of polyplexes in the spleen 4 h postinjection. In the quantitative analysis of polyplex uptake per cell, dendritic cells and monocytes exhibited the highest efficiency in taking up both non‐PEGylated and PEGylated polyplexes among diverse immune cell types, followed by macrophages (Figure 5G). When the overall contribution of each immune cell type in splenic uptake of the polyplexes was calculated by multiplying uptake efficiency per cell and percentages of each cell type (Figure S6, Supporting Information), B cells, which are abundant in the spleen, primarily contributed to the uptake (Figure 5H).

### 2.4 Application to Vaccines

**Figure 6:**
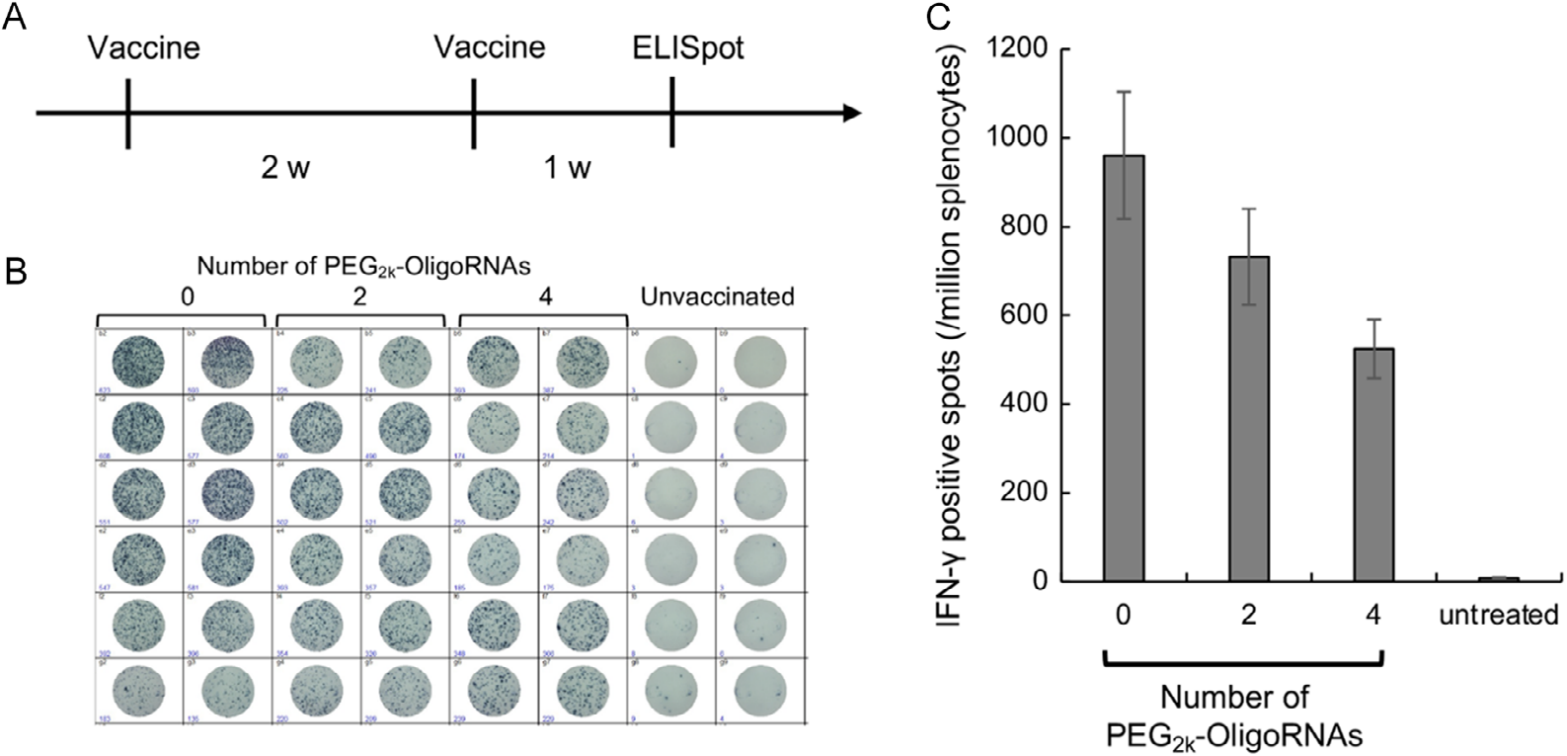
Vaccination using model antigen mRNA. Polyplexes loading *OVA* mRNA were injected twice every 2 weeks. An ELISpot assay was performed 1 week after the second dose. A) A vaccination schedule, B) appearance of an ELISpot plate, and C) the number of IFN‐γ positive spots, *n* = 6. Data are presented as the mean ± SEM.

Previous studies on mRNA vaccines have highlighted the potential of spleen‐targeting systems in cancer vaccines.^[^ ^32^, ^33^, ^34^, ^35^ ^]^ Thus, we assessed the potential benefits of the PEGylated mRNA polyplexes developed in this study for future biomedical applications, specifically by exploring their application in vaccination. To evaluate cellular immunity induction, we conducted Enzyme‐Linked ImmunoSpot (ELISpot) assays following the systemic injection of mRNA encoding ovalbumin (OVA), a model antigen. Polyplexes loaded with mRNA hybridized with 2 or 4 PEG_2k_–OligoRNAs elicited a substantial number of OVA‐reactive splenic cells, although the numbers were slightly lower than those observed after the delivery of non‐PEGylated polyplexes (**Figure** **6**). Notably, the polyplexes with 2 or 4 PEG_2k_–OligoRNAs may efficiently express antigen protein (Figure 4A–C) and type I IFN (Figure 4F) in the spleen. Type I IFN responses potentially enhance cellular immunity induction in mRNA vaccines by serving as an immunostimulatory adjuvant.^[^ ^32^, ^34^, ^36^ ^]^ These mechanisms could contribute to preserving high vaccination efficiency even after PEGylation. Potential safety advantages of PEGylated polyplexes over non‐PEGylated polyplexes include minimal expression of antigens and type I IFN responses in the lung (Figure 4A–C,G) and the absence of lung embolism (Figure 5D–F). Notably, ectopic antigen expression in non‐lymphatic tissues could trigger autoimmune reactions against the tissue.^[^ ^37^ ^]^ However, further studies are required to clarify the actual safety benefits of PEGylation in mRNA vaccines.

## 3 Conclusion

This study successfully achieved spleen‐selective delivery of mRNA polyplexes by precisely controlling polyplex PEG‐coating through an mRNA‐engineering approach. PEGylating polycations, an orthodox polyplex PEGylation approach, encounters difficulties in controlling the amount of PEG attached to the polyplexes, as PEGylated polycations can exist in both free and mRNA‐binding forms. In contrast, our mRNA PEGylation strategy ensured that nearly all added PEG‐OligoRNAs bound to mRNA and became integrated into the polyplexes, allowing for precise control of PEG amounts attached to the polyplexes. As observed in polyplexes prepared from PEGylated polycations,^[^ ^22^ ^]^ PEGylation using PEG–OligoRNAs hampered the cellular uptake and endosomal escape of the polyplexes (Figure 3B–D). However, the hybridization of PEG–OligoRNAs did not impair the protein translational process inside the cells, regardless of the number and lengths of the PEG strands (Figure 3E), establishing a valuable platform for polyplex PEGylation.

Notably, even slight variations in PEG amount and length profoundly impacted the physicochemical properties and biological functionalities of the polyplexes. The introduction of a small number of short PEG strands prevented the polyplexes from agglomerating in the bloodstream and subsequent lung deposition while maintaining mRNA delivery efficiency in the spleen (Figure 4). In contrast, minor increases in PEG–OligoRNA numbers impaired protein expression in the spleen, whereas non‐PEGylated polyplexes nonselectively provided protein expression in both the lung and spleen. These results underscore the advantages of fine‐tuned PEG coating in our approach to achieve organ‐selective delivery of mRNA polyplexes.

Tissue targeting mechanisms can be discussed at cellular and organ levels. At the cellular level, DC2.4 cells derived from dendritic cells exhibited enhanced efficiency of reporter protein expression after treatment with non‐PEGylated and PEGylated polyplexes compared to lung‐cancer‐derived A549 cells and hepatoma‐derived HuH‐7 cells (Figure S2, Supporting Information). This result suggests that the polyplexes may possess intrinsic capabilities to deliver mRNA efficiently into immune cells. In vivo evaluation revealed that B cells predominantly contributed to polyplex uptake in the spleen (Figure 5H). The contribution of phagocytes to overall polyplex accumulation in the spleen was lower than that of B cells despite their high activity of polyplex uptake (Figure 5G). This is attributed to the much smaller number of phagocytes in the spleen than B cells (Figure S6, Supporting Information). Collectively, abundance of immune cells in the spleen, efficiently taking up the polyplexes, might explain the mechanism behind the substantial accumulation of polyplexes in this organ. At organ level, non‐PEGylated polyplex immediately formed agglomerates in the blood circulation, presumably clotting the lung capillary (Figure 5), leading to considerable protein expression in the lung (Figure 4A–C). In contrast, optimally PEGylated polyplexes did not aggregate in the blood circulation, avoiding lung accumulation. Meanwhile, the spleen can physically entrap the nanoparticles with smaller sizes, i.e., over 100–200 nm, by the endothelial slits in splenic sinuses.^[^ ^38^ ^]^ Non‐PEGylated and PEGylated polyplexes forming a protein corona might exploit this organ‐level mechanism in their splenic accumulation, along with their potential cellular‐level tropism to the immune cells.

However, the cellular mechanism underlying efficient mRNA delivery of both non‐PEGylated and PEGylated polyplexes in immune cells remains unclear. A recent study on LNPs reported that the composition of LNPs influenced the types of protein corona attached to them, determining the target tissues of the LNPs.^[^ ^9^ ^]^ Similarly, the protein corona attached to the PEGylated polyplexes might explain their selectivity for the spleen. Further clarification of these mechanisms may aid in designing organ‐selective mRNA polyplexes in the future. In addition, future therapeutic investigations will also require safer and more functional polycations, while this study employed LPEI, a gold standard polymer, for proof of concept. By enabling spleen‐specific mRNA delivery, this study has the potential to expand the applications of mRNA polyplexes, such as in cancer vaccines, though further studies are necessary to assess the therapeutic utility of our systems.

## 4 Experimental Section

4.1

4.1.1

##### Hybridization of PEG‐OligoRNAs to mRNA

mRNA encoding *fLuc* and *OVA* were purchased from Trilink (San Diego, CA, USA). The 2 and 12 kDa PEG were conjugated to the 5' end of RNA oligonucleotides by Gene Design (Osaka, Japan). In designing PEG–OligoRNA sequences, the NUPACK software (https://www.nupack.org) was used to predict the secondary structure of mRNA. The sequences of 7 RNA oligonucleotide species (#1–#7) for *fLuc* mRNA and 4 RNA oligonucleotide species (#8–#11) for *OVA* mRNA are listed in Table S2, Supporting Information. We selected #4 for 1 PEG–OligoRNA in fLuc, #2 and #6 for 2 PEG–OligoRNAs in fLuc, #1, #3, #5, and #7 for 4 PEG–OligoRNAs in fLuc, #1–#7 for 7 PEG–OligoRNAs in fLuc, #8 and #10 for 2 PEG–OligoRNAs in OVA, and #8–#11 for 4 PEG–OligoRNAs in OVA. mRNA was mixed with PEG–OligoRNAs at a molar ratio of mRNA to each oligo RNA of 1. The mixture containing 150 mM NaCl was heated at 65 °C for 5 min followed by cooling to 30 °C in 10 min for hybridization. Hybridization efficiency was evaluated by gel electrophoresis, using tris borate EDTA polyacrylamide gel with an acrylamide concentration of 10% for PEG_2k_–OligoRNAs and 6% for PEG_12k_–OligoRNAs. The gels stained with SYBR Green II (5771A, Takara Bio, Tokyo Japan) were visualized using a Gel Doc EZ (BioRad, Hercules, CA, USA).

##### Polyplex Preparation and Characterization

An equal volume of mRNA and LPEI (in vivo jetPEI, polyplus, Illkirch, France) solutions were mixed after adjusting the concentration of each solution to obtain the indicated N/P ratios. The binding of mRNA to LPEI was evaluated by 1% agarose gel electrophoresis, followed by SYBR Green II staining and visualization using a Gel Doc EZ. The size and ζ potential of each sample were measured by DLS and phase analysis light scattering (PALS) with a Möbius (Waters Corporation, Santa Barbara, CA, USA). Samples were equilibrated at 25 ± 0.1 °C for 5 min prior to the measurements. DLS data was collected with a 10 s acquisition time and 10 acquisitions per measurement. PALS measurement was performed by applying a voltage of 2.5 V at a frequency of 10 Hz for 15 s, and ζ potential was calculated using the Smoluchowski equation. All measurements were repeated three times.

##### In Vitro Experiments

DC2.4 (Merck Millipore, Burlington, MA), A549 (RIKEN Cell Bank, Tsukuba, Japan), and HuH‐7 cells (RIKEN Cell Bank) were cultured as previously described.^[^ ^24^, ^39^, ^40^ ^]^ For the fLuc expression tests, the cells were seeded into 96‐well plates at 10 000 cells/well and incubated for 24 h. Polyplexes containing 100 ng mRNA were added to each well. In the experiment as shown in Figure 3A, after incubation for 24 h, cells were lysed with passive lysis buffer (E1941, Promega, Madison, WI, USA), and added with luciferase substrate (E151A, Luciferase Assay System, Promega) for luminescence measurement using Lumat^3^ LB9508 luminometer (Berthold technologies, Bad Wildbad, Germany). In the experiments for Figure S2 and S4, Supporting Information, the cells were lysed and colorized with the ONE‐Glo Luciferase Assay System (Promega), followed by luminescence measurement using a GloMax luminometer (Promega). For the inhibition assay of cellular uptake routes, the cells were preincubated with 10 μM chlorpromazine (Fujifilm Wako, Osaka, Japan), 100 μM genistein (Fujifilm Wako), or 0.5 μM cytochalasin D (Fujifilm Wako) for 30 min before adding the polyplexes. For the cellular uptake tests, *fLuc* mRNA was labeled with Cy3 using *Label* IT Tracker Intracellular Nucleic Acid Localization Kit (Mirus, Madison, WI, USA). DC2.4, A549, and HuH‐7 cells were seeded into 12‐well plates at 100 000 cells/well and incubated for 24 h. Polyplexes containing 100 ng of Cy3‐labeled mRNA were added to each well. After incubation for 1 h, the cells were detached with trypsin–EDTA and washed three times with PBS, followed by fixation with 4% paraformaldehyde for 15 min at 4 °C. The cells were further washed with PBS, and fluorescence intensity from the single cells was measured by a flow cytometer RF‐500 (Sysmex, Hyogo, Japan). For the cellular uptake tests by qPCR, DC2.4 cells were seeded into 12‐well plates at 200 000 cells/well and incubated for 24 h. Polyplexes containing 2 μg of *fLuc* mRNA were added to each well. After incubation for 24 h, total RNA was extracted from the cells and purified using RNeasy Mini Kit (Qiagen, Hilden, Germany), followed by reverse transcription using ReverTra Ace qPCR RT Master Mix with gDNA Remover (TOYOBO, Osaka, Japan). qPCR was performed using a Mic qPCR Cycler (Bio Molecular Systems, Queensland, Australia). The fLuc sequence was detected using a primer pair (forward: CAAGAAGGGCCTGCAGAAGA, reverse: GCTGGTCACGAAGGTGTACA) and SYBR Green qPCR Master Mix (Roche, Basel, Switzerland). Mouse actin‐beta  (4 352 663, Applied Biosciences) was used for detecting *β‐Actin*. For the membrane destabilization test, DC2.4 cells were seeded into 96‐well plates at 20 000 cells/well and incubated for 24 h. The pH of the medium was adjusted with hydrochloric acid. Polyplexes containing 400 ng of *fLuc* mRNA were added to each well. After incubation for 24 h, the supernatant was transferred to fresh 96‐well plates and diluted four times with PBS, followed by the quantification of LDH using Cytotoxicity LDH Assay Kit‐WST (Dojindo, Kumamoto, Japan) and a plate reader Infinite 200 PRO (Tecan, Mannedorf, Switzerland). For the cell‐free translation test, polyplexes containing 400 ng of *fLuc* mRNA were translated using Rabbit Reticulocyte Lysate System (Promega) at 30 °C for 1.5 h. The solution was added with luciferase substrate in Luciferase Assay System (Promega) for luminescence measurement using a Glomax luminometer.

##### Measurement of Luciferase Expression and mRNA Amount in Mouse Tissues

All animal experiments in this study were approved by the animal care and use committees in Kyoto Prefectural University of Medicine (M2021‐518) and the Innovation Center of NanoMedicine, Kawasaki Institute of Industrial Promotion (A17‐009‐1, A20‐018‐4). BALB/c mice (female, 7 weeks old, Charles River Laboratories Japan, Yokohama, Japan) were injected with 200 μL of a polyplex solution containing 5 μg of mRNA via their tail vein. And, 4 h after the injection, the lung, spleen, and liver were excised and homogenized using a Micro Smash MS‐100 (Tomy Digital Biology, Tokyo, Japan), followed by luminescence measurement using a Lumat^3^ LB9508 luminometer. The luminescence values were standardized based on protein amount quantified using the Micro BCA Protein Assay Kit (Thermo Fisher Scientific). For qPCR, total RNA in the homogenates was purified using the RNeasy Mini Kit (Qiagen, Hilden, Germany), followed by reverse transcription using a ReverTraAce with gDNA remover kit (TOYOBO, Osaka, Japan). qPCR was performed as described earlier using the Taqman Gene Expression Assay system (Thermo Fisher Scientific) for detecting *IL‐6* (Mm00446190_m1), *IFN‐β* (Mm00439552_s1), and *β‐Actin* (4 351 315).

##### Imaging of Living Mice and Mouse Tissues

BALB/c mice (female, 7 weeks old) were injected with 200 μL of a polyplex solution containing 5 μg of mRNA via their tail vein. At 4 h postinjection, the mice were intraperitoneally injected with 150 mg kg^−1^ of VIvoGlo Luciferin (Promega), followed by whole‐body luminescence imaging using an in vivo imaging system (IVIS) Spectrum (Perkin Elmer, Waltham, MA, USA). At the same time point, the lung, spleen, liver, and kidneys were excised from the mice and soaked in a 15 mg mL^−1^ luciferin solution for 10 min to perform the luminescence imaging of individual organs. For the intravital imaging of polyplexes in the bloodstream, *fLuc* mRNA was labeled with Cy5 using the *Label* IT Tracker Intracellular Nucleic Acid Localization Kit (Mirus, Madison, WI, USA). BALB/c mice (female, 7 weeks old) were anesthetized with isoflurane and placed on the microscope stage of the A1R^+^ in vivo confocal microscope (Nikon, Tokyo, Japan). The vein in the earlobe dermis was set as the region of interest. Fluorescence images were recorded in video mode after injecting 200 μL of a polyplex solution containing 20 μg of Cy5‐labeled mRNA via their tail vein.

For tissue imaging, the mouse lung was frozen 30 min after intravenous injection of polyplexes loading 5 μg of Cy5 labeled mRNA. The 4 μm thick sections were prepared and stained with 4',6‐diamidino‐2‐phenylindole (DAPI) Fluoromount‐G (Southern Biotech, Birmingham, AL, USA), followed by observation using a fluorescence microscopy (BZ‐X810, Keyence, Osaka, Japan).

##### Toxicity Evaluation

BALB/c mice (female, 7 weeks old) were injected with 200 μL of a polyplex solution containing 5 μg of *fLuc* mRNA via their tail vein. At 24 h postinjection, blood was collected via the inferior vena cava and centrifuged to obtain the plasma. The concentration of alanine aminotransferase (ALT), aspartate ALT, LDH, blood urea nitrogen, and creatinine was measured using a Dri‐Chem 7000IZ (Fujifilm, Tokyo, Japan).

##### Flow Cytometry Using Mouse Splenocytes

BALB/c mice (female, 7 weeks old) were injected with 200 μL of a polyplex solution containing 5 μg of Cy5‐labeled mRNA via their tail vein. At 4 h postinjection, the spleen was harvested from the mice and dissociated using a metal mesh, followed by filtration through a 40 μm cell strainer. Red blood cells were lysed with BD Pharm Lyse (BD Biosciences, Franklin Lakes, NJ, USA). The obtained splenocytes were incubated with anti‐mouse CD16/32 antibody (BioLegend, San Diego, CA, USA) on ice for 10 min and then the antibody cocktails in the dark for 30 min. The antibody cocktail for T and B cells contained fluorescein isothiocyanate anti‐CD3ε (145‐2C1) and Brilliant Violet 510 anti‐CD19 (6D5) (BioLegend). The antibody cocktail for myeloid cells contained phycoerythrin (PE) anti‐CD11b (M1/70), Brilliant Violet 510 anti‐CD11c (N418), Brilliant Violet 421 anti‐F4/80 (BM8), Alexa Fluor 488 anti‐Ly6C (HK1.4), and PE/Cy7 anti‐Ly6G (1A8) (BioLegend). After the incubation, the cells were washed twice and stained with DAPI (Thermo Fisher Scientific, Waltham, MA, USA) before measurement using the LSRFortessa X‐20 (BD Biosciences). More than 100 000 cells were collected for each sample. Single cells were gated based on forward scatter (FSC)‐H versus FSC‐A, and then viable cells were gated as DAPI^−^. The viable cells were further gated into the following subpopulations: B cells (CD19^+^), T cells (CD19^−^ CD3^+^) DCs (CD11b^+^ CD11c^+^), neutrophils (CD11b^+^ CD11c^−^ Ly6G^+^), eosinophils (CD11b^+^ CD11c^−^ Ly6G^−^ F4/80^+^ Ly6C^Int^ SSC^hi^), monocytes (CD11b^+^ CD11c^−^ Ly6G^−^ F4/80^+^ Ly6C^hi^), and macrophages (CD11b^+^ CD11c^−^ Ly6G^−^ F4/80^+^ Ly6C^int/neg^ SSC^lo^).

##### Mouse Vaccination

Polyplexes loading PEGylated *OVA* mRNA (5 μg) were injected twice every 2 weeks. One week after the second dose, the spleen was collected for ELISpot assay using anti‐IFN‐γ ELISpot PLUS kits (Mabtech, Nacka Strand, Sweden). The splenocytes were seeded at 500 000 cells/well for an overnight incubation in 10 nmol of OVA peptide (257–264) (Peptide Institute, Osaka, Japan), followed by horseradish peroxidase staining according to the manufacturer's protocol and spot counting using an ELISpot plate reader (AID GmBH, Germany).

##### Statistical Analysis

In physicochemical analyses (Figure 2 and Table S1, Supporting Information), data are presented as the mean ± SD. In the other experiments, data are presented as the mean ± standard error of the mean (SEM). Sample sizes were described in figure legends. Statistical analyses were performed by ANOVA followed by Dunnett's test or Tukey's post hoc test (see figure legends for post hoc tests) using Kaleida Graph (Hulinks, Tokyo, Japan). P values are described in the figures or expressed as *p < 0.05, **p < 0.01, and ***p < 0.001.

## Conflict of Interest

S.U. is a chief medical officer of Crafton Biotechnology.

## Supporting information

Supplementary Material

## Data Availability

The data that support the findings of this study are available from the corresponding author upon reasonable request.
