# Supplementary material for: Poly(ethylene Glycol) (PEG)–OligoRNA Hybridization to mRNA Enables Fine‐Tuned Polyplex PEGylation for Spleen‐Targeted mRNA Delivery
Source: Small Sci. 2024 Feb 22;4(4):2300258. doi: 10.1002/smsc.202300258 (PMC11935287; doi:10.1002/smsc.202300258)
Supplement: Supplementary file 1 — Supplementary Material [file SMSC-4-2300258-s001.pdf]

Supporting Information

PEG-OligoRNA Hybridization to mRNA Enables Fine-Tuned Polyplex PEGylation for Spleen-Targeted mRNA Delivery

*Miki Suzuki[#], Yuki Mochida[#], Mao Hori, Akimasa Hayashi, Kazuko Toh, Theofilus A. Tockary, Xueying Liu, Victor Marx, Hidetomo Yokoo, Kanjiro Miyata, Makoto Oba, and Satoshi Uchida**

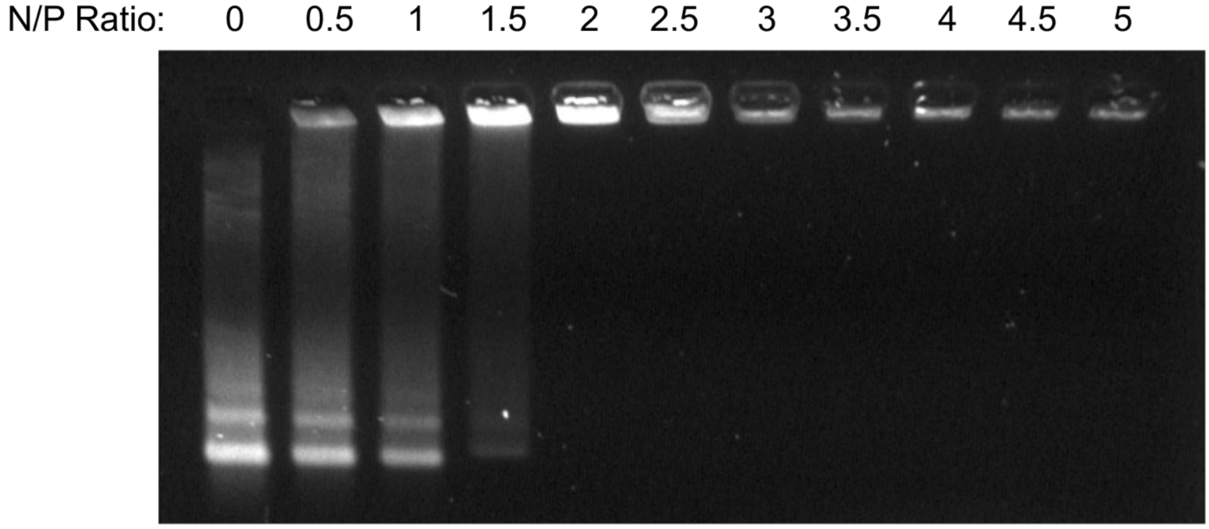

Supplementary Figure S1. Gel electrophoresis of LPEI/mRNA polyplexes at different N/P ratios.

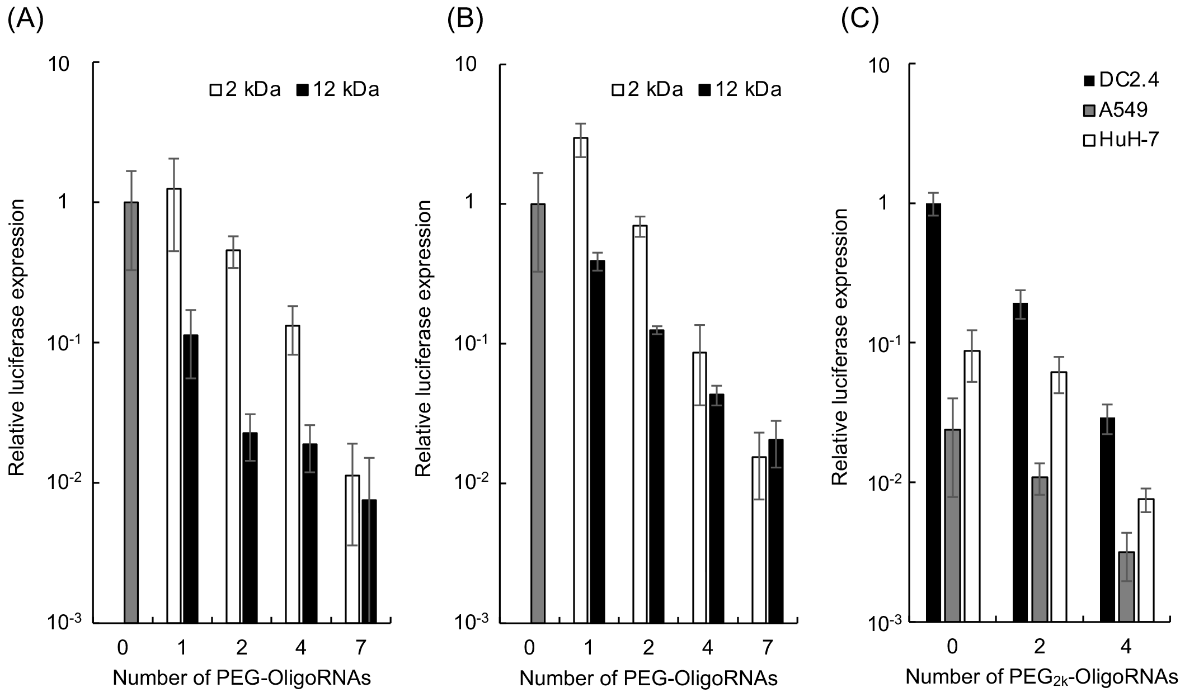

Supplementary Figure S2. fLuc expression efficiency in cell lines derived from lung and liver cancers. (A) Lung cancer-derived A549 cells. (B) Liver cancer-derived HuH-7 cells. (C) Expression efficiency in DC2.4, A549, and HuH-7 cells after treatment with non-PEGylated polyplexes. fLuc expression efficiency was quantified after 4 h of polyplex treatment. The data are standardized with those from non-PEGylated polyplexes of A549 cells (A), HuH-7 cells (B) and DC2.4 cells (C). The data in (C) are reconstructed from **Supplementary Figure S2** (A, B) and **Supplementary Figure S4** (C). Note that all of these experiments were performed simultaneously using the same conditions. $n = 6$. Data are presented as the mean ± standard error of the mean (SEM).

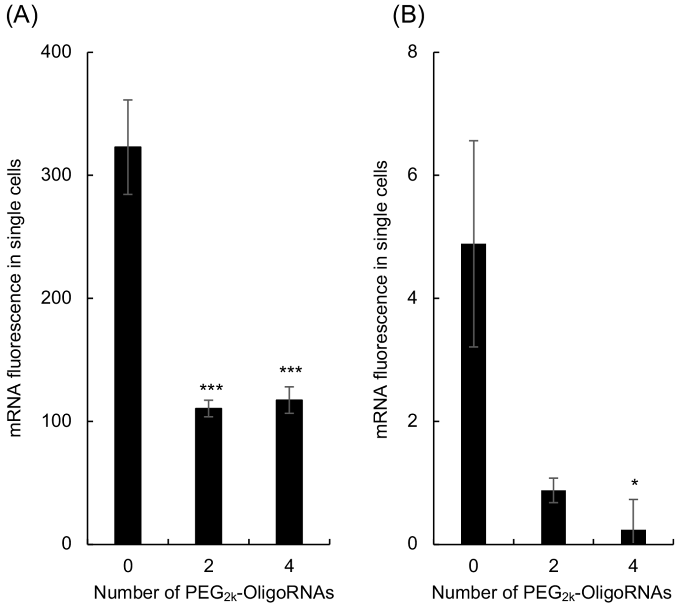

Supplementary Figure S3. Cellular uptake efficiency of polyplexes in cell lines derived from lung and liver cancers. (A) Lung cancer-derived A549 cells. (B) Liver cancer-derived HuH-7 cells. Data are shown after subtraction of fluorescence intensity from untreated cells. $n = 3$ or 4. Data are presented as the mean ± standard error of the mean (SEM). Statistical analyses were performed by analysis of variance (ANOVA) followed by Dunnett's test. *$p < 0.05$, ***$p < 0.001$ vs. non-PEGylated polyplexes.

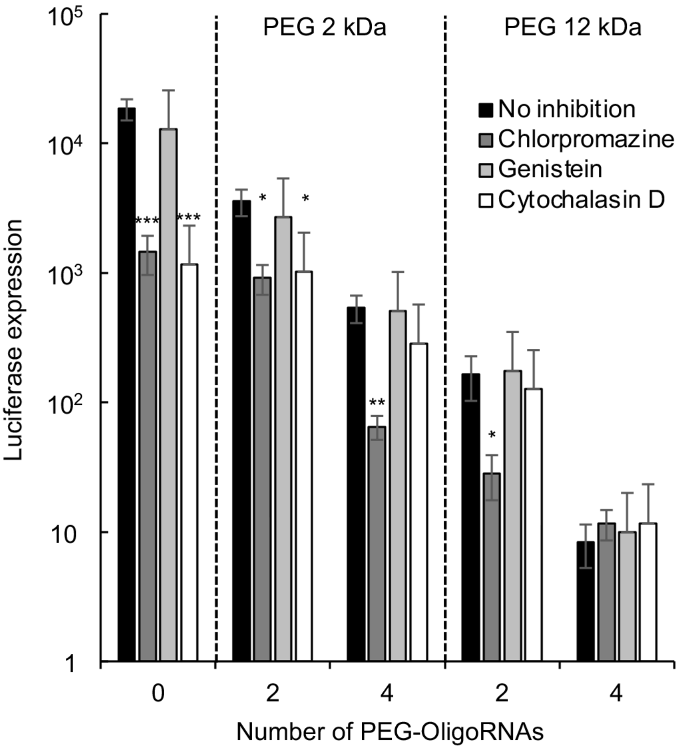

Supplementary Figure S4. Cellular uptake pathways of the polyplexes. The influence of endocytosis inhibitors on mRNA delivery efficiency in DC2.4 cells was evaluated using chlorpromazine for inhibiting clathrin-mediated endocytosis, genistein for inhibiting caveolae-mediated endocytosis, and cytochalasin D for inhibiting phagocytosis and micropinocytosis. Cells were treated with one of these inhibitors and then polyplexes, followed by fLuc measurement 4 h after polyplex treatment. $n = 6$. Data are presented as the mean ± standard error of the mean (SEM). Statistical analyses were performed by analysis of variance (ANOVA) followed by Dunnett's test. *$p < 0.05$, **$p < 0.01$, ***$p < 0.001$ vs. no inhibition.

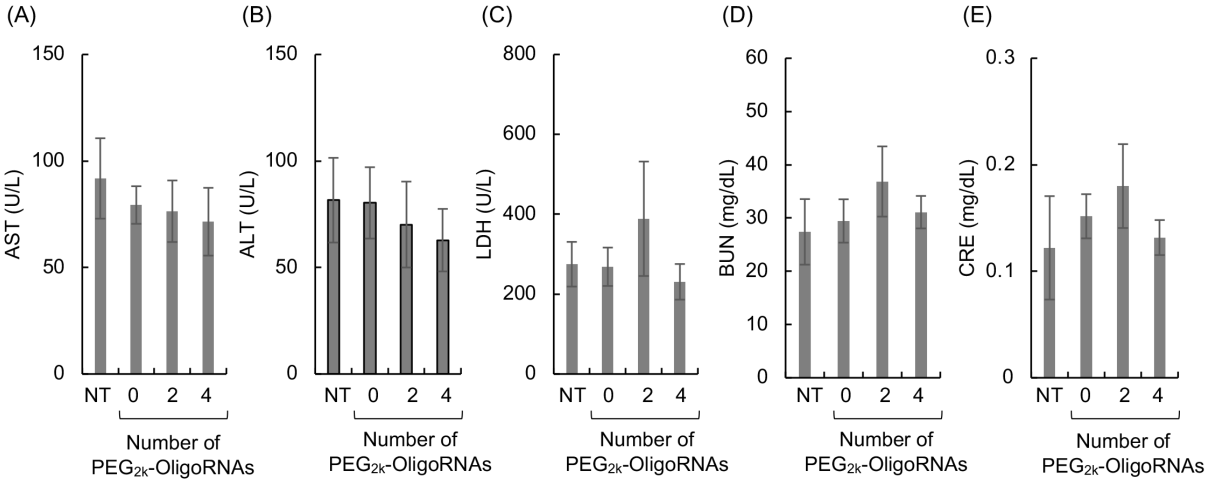

Supplementary Figure S5. Toxicity assays. Blood was collected 24 h post-injection of polyplexes loading 5 µg of *fLuc* mRNA. (A) Aspartate aminotransferase (AST). (B) Alanine aminotransferase (ALT). (C) Lactate dehydrogenase (LDH). (D) Blood urea nitrogen (BUN). (E) Creatinine (CRE).

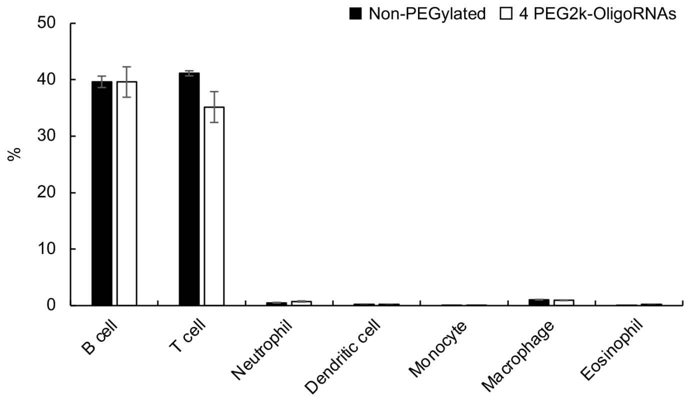

Supplementary Figure S6. Percentages of each cell type in flow cytometry performed for Figure 5G, H.

Supplementary Table S1. Polydispersity index (PDI) of the polyplexes.

Number of PEG-OligoRNAs	0	1	2	4	7	1	2	4	7
PEG Mw (kDa)		2	2	2	2	12	12	12	12
PDI	0.121 ± 0.004	0.149 ± 0.000	0.148 ± 0.015	0.135 ± 0.013	0.137 ± 0.007	0.127 ± 0.004	0.110 ± 0.011	0.098 ± 0.016	0.134 ± 0.011

Supplementary Table S2

Number	Target gene	Position*	Sequence
#1	*fLuc*	17	GGGGCCCUUCUUGAUGU
#2	*fLuc*	316	UCGUUGUAGAUGUCGUU
#3	*fLuc*	401	CACGUUCAGGAUCUUCU
#4	*fLuc*	694	AUGGCGGUGUCGGGGAU
#5	*fLuc*	882	GGGUGCUCUUGGCGAAG
#6	*fLuc*	1057	UUGUCGUCGCCCUCGGG
#7	*fLuc*	1315	UACUUGAUCAGGCUCUU
#8	*OVA*	252	GGUUCAGGAUGUCCCGC
#9	*OVA*	563	GUCCUCGUCCUUGAAGG
#10	*OVA*	777	GCUUCUCGAAGUUGAUG
#11	*OVA*	1103	GGCGAUGUGCUUGAUGC

* Positions of the first bases in mRNA hybridized with PEG-OligoRNAs are presented as nucleotide numbers from mRNA
